# A mechanokinetic actomyosin model predicts different orthophosphate sensitivities of force and ATP turnover rate during isometric muscle contraction

**DOI:** 10.3389/fphys.2025.1659772

**Published:** 2025-10-10

**Authors:** Alf Månsson

**Affiliations:** Department of Chemistry and Biomedical Sciences, Faculty of Health and Life Science, Linnaeus University, Kalmar, Sweden

**Keywords:** inorganic phosphate, isometric contraction, ATP turnover rate, force, number of attached cross-bridges

## Abstract

The release of the ATP hydrolysis product, orthophosphate (Pi), from the myosin active site, together with force-generating structural changes, is central to actomyosin energy transduction, but the temporal order of these events remains unclear. A range of data, interpreted using simple kinetic schemes (that do not account for varying cross-bridge strains) suggests that force generation is closely associated with the attachment of the myosin head to actin, preceding Pi-release. However, the addition of a branched pathway to the kinetic scheme is needed to account for the lower sensitivity of the isometric ATP-turnover rate to Pi compared with that of force. In contrast, a branched pathway does not appear necessary if the data are analyzed using a mechanokinetic model that incorporates the myosin strain distribution. Here, we corroborated this idea using a model in which Pi-release from the active site precedes the force-generating power-stroke. We explain the effect based on two components underlying the reduction in isometric force with increased [Pi]. The larger component arises from pre-power-stroke cross-bridges with high large elastic strain, whereas the smaller component results from cross-bridges attaching with low elastic strain. Because only the latter myosin heads undergo ATPase cycles, force exhibits greater Pi-sensitivity than ATPase activity. Changes in model parameter values that minimize the width of the cross-bridge strain distribution do not eliminate the difference in Pi-sensitivity between isometric force and ATPase. Such changes, including reduced actin affinity in a pre-power-stroke state, also lead to a proportional reduction in isometric force and in the number of attached cross-bridges with increased [Pi]. In conclusion, our data suggest that a mechanokinetic model explains the combined changes in isometric force, ATPase activity, and the number of attached cross-bridges with varied [Pi] more directly than apparently simpler kinetic schemes. A central feature of these results is the explicit demonstration of two components of isometric force with different physiological roles.

## 1 Introduction

The generation of force and motion by cyclic interactions between myosin motors and actin filaments is central in eukaryotic biology (Heissler and Sellers, 2016). This includes muscle contraction as the most apparent manifestation and also includes movement and internal organization of non-muscle cells, functioning of the immune system, and cell signaling. Increasing evidence indicates that disturbances in actin–myosin function play key roles in a range of diseases such as cardiac conditions (hereditary cardiomyopathies) (Yotti et al., 2019), spasticity (Gyimesi et al., 2020), cancer (Kenchappa et al., 2025), drug abuse (Young et al., 2016), and malaria (Moussaoui et al., 2020; Vahokoski et al., 2022). Consequently, related drug development efforts (Trivedi et al., 2020) aim at different myosin classes as drug targets of interest. This is most prominent for compounds that are potentially active against cardiomyopathies and other heart conditions (reviewed by Lehman et al., 2022). Particularly, a growing number of small-molecule compounds have been synthesized and tested with the aim to treating or alleviating symptoms in hypertrophic cardiomyopathy and heart failure. One of these drugs was recently approved by the regulatory authorities for clinical use in obstructive hypertrophic cardiomyopathy (Spertus et al., 2021).

It is of critical importance to understand the details of effective actin–myosin energy transduction not only for fundamental insights into this central biological process but also for understanding the abovementioned diseases and enabling effective drug development. Detailed insights have been gained through intense studies over decades (reviewed by Geeves, 2016; Robert-Paganin et al., 2020; and Rassier and Månsson, 2025). However, understanding of the most central events in the cycle—from myosin attachment to actin, through Pi-release from the active site, to the associated force-generating structural changes and increased actin–myosin affinity—remains limited. Specifically, there is a wide range of models for the temporal relationship between the release of the ATP-hydrolysis product orthophosphate (Pi, inorganic phosphate) from the active site of myosin and the main force and motion-generating lever arm swing (the power-stroke) (reviewed by Månsson et al., 2015; Stehle and Tesi, 2017; Debold, 2021; Månsson et al., 2023; Kaya, 2025; and Rassier and Månsson, 2025). There are models assuming Pi-release from the active site distinctly before, concomitant with, or after the power-stroke (reviewed by Månsson et al., 2015). However, these models come in more complex types with different branched schemes (Linari et al., 2010; Malnasi-Csizmadia and Kovacs, 2010; Debold et al., 2011; Debold et al., 2013; Governali et al., 2020; Scott et al., 2021; Marang et al., 2025) and with postulated secondary Pi-binding sites outside the active site (Moretto et al., 2022). Finally, there are models that favor loose coupling between Pi-release and the power-stroke, i.e., assuming that either order of the two events is possible depending on the conditions (Malnasi-Csizmadia and Kovacs, 2010; Caremani et al., 2013; Caremani et al., 2015).

Despite extensive studies, it has not been possible to conclusively distinguish between the abovementioned models. Not even recent insights gained from cryo-EM images of both pre- and post-power-stroke states of myosin (Klebl et al., 2025) have resolved this issue. Some of the experimental studies aiming to elucidate the coupling between Pi-release and force generation have been performed with isolated actin filaments and soluble myosin motor fragments (e.g., heavy meromyosin and myosin sub-fragment 1) using transient biochemical solution kinetics (Muretta et al., 2015; Rohde et al., 2017). Such studies are generally interpreted using simple kinetic schemes that do not consider a distribution of elastic strains of each biochemical actin–myosin state (i.e., a state with either ATP or different combinations of products in the active site). This is well-justified by the lack of fixed geometrical arrangements between the interacting proteins. However, simple kinetic schemes are also often used to interpret experimental results from isometrically contracting muscle cells or myofibrils, i.e., muscle preparations where the distance between the insertion points in the recording apparatus is constant (Kawai and Halvorson, 1991; Dantzig et al., 1992; Ranatunga, 1999; Tesi et al., 2000; Stehle, 2017; Kawai et al., 2021; Stehle, 2024). This is usually justified by the principle of simplicity (Occam’s razor), coupled with the assumption that the attached myosin motors during isometric contraction experience a relatively narrow range of strains (*cf.* Linari et al., 2010). However, this view has been questioned recently (Månsson, 2025), and we consider this issue in greater detail below.

**FIGURE 1:**
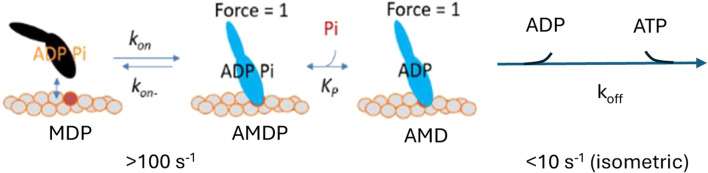
Simple kinetic scheme found to account for a range of experimental data at varied Pi. A, actin; M, myosin; D, ADP; and P, Pi.

Several experimental results at varied [Pi] from solution biochemistry (Muretta et al., 2015; Rohde et al., 2017) and studies of isometrically contracting muscle fibers (Kawai and Halvorson, 1991; Dantzig et al., 1992; Ranatunga, 1999; Tesi et al., 2000; Tesi et al., 2002; Stehle, 2017; Kawai et al., 2021; Stehle, 2024), along with some single-molecule studies (Woody et al., 2019), are consistent with a simple kinetic scheme similar to that in Figure 1.

Such a scheme accounts well for the Pi-dependence of both the isometric force and the number of attached cross-bridges during isometric contraction (Caremani et al., 2008; Linari et al., 2010). However, the appreciably smaller effect of varied [Pi] on the ATP turnover rate than on tension during steady-state isometric contraction in fast rabbit psoas muscle is not accounted for. Therefore, Linari et al. (2010) introduced a branched pathway, assuming that a fraction of the myosin heads detaches from actin in a post-force-generation state with Pi still at the active site. With an appropriate selection of rate constants, this detachment pathway permits high ATP turnover at elevated [Pi] while maintaining a pronounced decrease in tension (Linari et al., 2010), thereby explaining the small effect of increased Pi on the ATP turnover rate. Another type of branched pathway with detachment induced by Pi-binding to a post-power-stroke state has also been introduced to account for the contractile phenomena beyond isometric contraction (Debold et al., 2011; Debold et al., 2013; Scott et al., 2021; Marang et al., 2025). However, also in the latter case, the branched model was found to better distinguish quantitatively different effects of increased [Pi] on isometric force and isometric ATPase than another model assuming that Pi-rebinding leads to a power-stroke reversal (Marang et al., 2025).

Mechanokinetic models differ from kinetic schemes by explicitly taking into account the distribution of elastic cross-bridge strains and the associated range of free energy levels and rate constants for each biochemical actomyosin state. Such models, rather than simple kinetic schemes, are necessary to interpret experiments involving changes in muscle length (Huxley, 1957; Hill, 1974; Eisenberg and Hill, 1978; Eisenberg et al., 1980) (reviewed by Rassier and Månsson, 2025). It is also interesting to note that models of this type (Smith, 2014; Månsson, 2021; and Månsson, 2025) may provide different predictions for the effects of varied [Pi] on isometrically contracting muscle than kinetic schemes that appear to be similar. We used such a mechanokinetic model recently (Månsson, 2025) to obtain predictions for the tension changes upon jumps in [Pi] (Pi-transients) and the rate of redevelopment of force from the zero level at varied [Pi]. While single exponential Pi-transients, as observed experimentally, were not predicted by this model, the transients were appreciably closer to single exponentials than those predicted by a similar kinetic scheme. Moreover, a previous mechanokinetic model of a similar type (Smith, 2014) appeared to have been even more successful in that regard. Notably, the latter model and a more recent mechanokinetic model (Månsson, 2021) predicted an appreciably lower effect of increased [Pi] on the isometric ATP turnover rate than isometric force without the introduction of a branched pathway. However, in view of the continued interest in branched models (Marang et al., 2025), it is important to revisit this problem.

To that end, we build on our recent analysis of Pi-transients and force-redevelopment during isometric contraction (Månsson, 2025). We show that this model (Månsson, 2025), like similar mechanokinetic models in previous work, predicts an appreciably smaller effect of increased [Pi] on the isometric ATPase than on the isometric tension. We then elucidate the basis for this result based on changes in cross-bridge distribution at varied Pi. In the Discussion section, we argue for the importance of using mechanokinetic models rather than simple kinetic schemes to interpret events during isometric contraction. We also consider the physiological roles of the two distinct components of isometric force that are identified in our analysis.

## 2 Materials and methods

**FIGURE 2:**
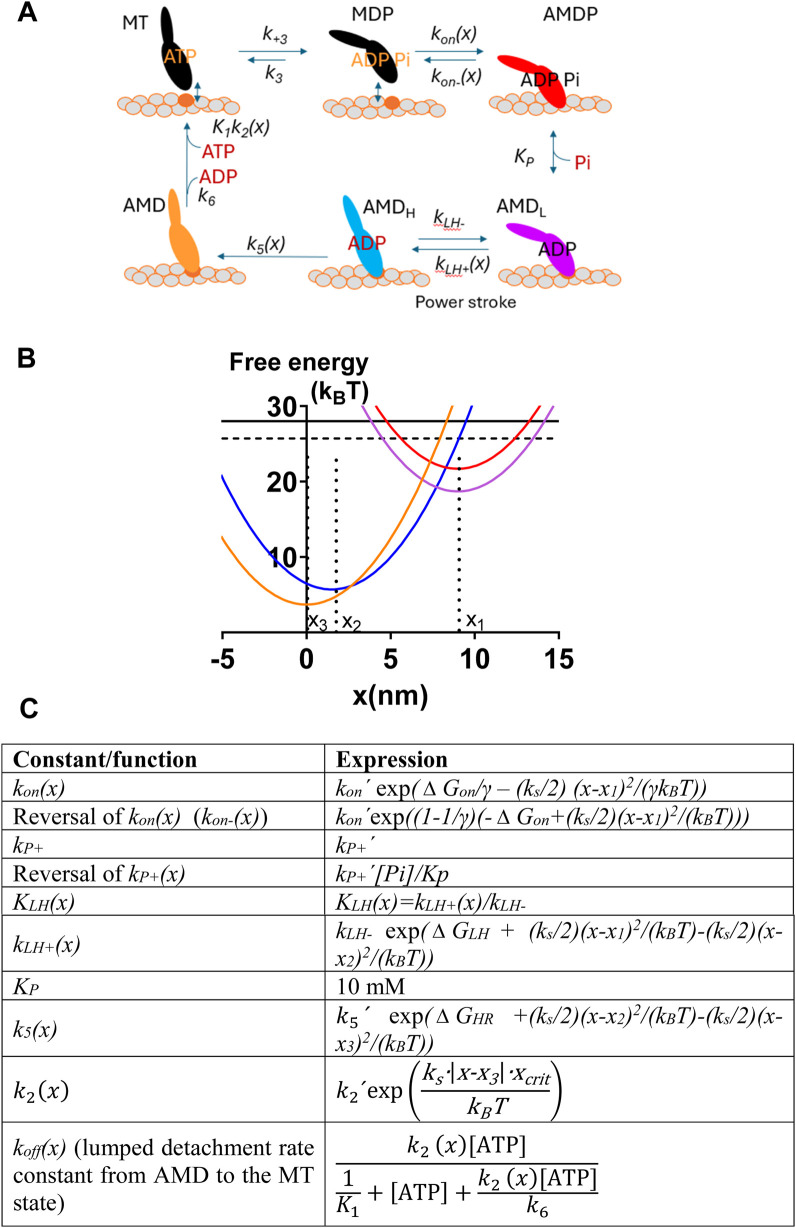
Mechanokinetic model. **(A)** Myosin (M) cross-bridge states that are either detached (black) from actin (“A”) or attached in different stereospecifically bound states where the color code (red, purple, blue, and orange) indicates the structural and/or biochemical similarity. Each state exists at different elastic strains. Myosin has either MgATP (T), MgADP (D), both MgADP and Pi (P), or no substrate or product in the active site. Labeling of the states as used below is indicated. The subscripts _L_ and _H_ refer to low and high force, respectively. The AMD_L_ to AMD_H_ transition is the main force-generating transition, the power-stroke. The argument (*x*) indicates the strain-dependence of rate constants. **(B)** Free energy of different states plotted against the position coordinate *x*. The color code corresponds to that given for the different states in A. The parameters *x*_*1*_, *x*_*2*_, and *x*_*3*_ are indicated in relation to the minima of the corresponding free energy diagrams. **(C)** *X* -dependence of the rate and equilibrium constants in the model in A–B. Notably, in some cases, the rate constants have one given, constant value for all *x*. The parameter values in the equations in the right column are given in Supplementary Tables S1 and S2, unless otherwise specified in the text. The lumped rate constant *k*_*off*_ *(x)* (Persson et al., 2013) is independent of MgADP because we assume that MgADP = 0 mM. MgATP is abbreviated as ATP in C. The figure is modified from Månsson (2025) for clarifications and to accommodate changes in the optimal parameter value settings.

**FIGURE 3:**
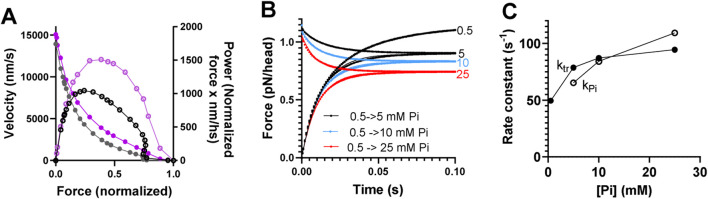
Predictions of mechanokinetic model. **(A)** Simulated force–velocity relationship and power at 0.5 mM Pi (black) compared to experimental data (purple) (Månsson et al., 1989) from mammalian muscle at 30 °C. Velocity (in nm per half-sarcomere/s; nm/s) is shown by closed symbols (left vertical axis), and power (velocity x normalized force; units nm/s) is shown by open symbols (right vertical axis). Experimental or simulated data are connected by lines for clarity. **(B)** Simulated data for the redevelopment of force from 0 at varied [Pi] (mM) are indicated, and Pi-transients starting at 0.5 mM Pi and ending at the Pi-levels (mM) are indicated. **(C)** Rate constants for the redevelopment of force (*k*_*tr*_) and Pi-transients (*k*_*Pi*_) vs. [Pi]. The rate constants were derived from the best fits to single exponential functions in B despite the Pi-transients being biphasic with a small fast component. No experimental data were given for comparison in B and C due to variability in the literature. However, note that the effects observed are within the experimental ranges. The data were reproduced with modifications from Månsson (2025).

**FIGURE 4:**
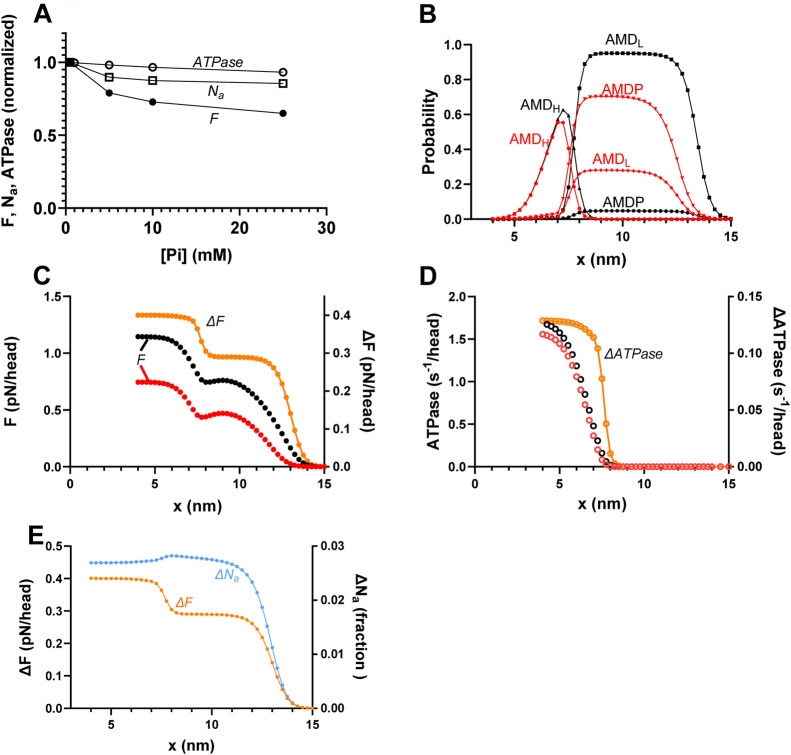
Predicted effects of varied [Pi] on the force (*F*), number of attached cross-bridges (*N*_*a*_), and ATP turnover rate (*ATPase*) during isometric contraction along with underlying mechanisms. **(A)** *F*, *ATPase*, and *N*_*a*_ vs. [Pi] for the standard model parameter values (Supplementary Tables S1, S2) summing up to the data for [Pi] = 0.5 mM. **(B)** Probability distribution of cross-bridge states (summing up to 1 for each *x* value) during steady-state isometric contraction of the model in Figure 2 at [Pi] = 0.5 mM (black) and 25 mM (red). The figure is similar to that previously shown by Månsson (2025). **(C)** Integral of force vs. *x* (left vertical axis) with integration starting at *x* = 15 nm and running in the negative *x*-direction at 0.5 mM (black) and 25 mM (red) Pi. Orange (right vertical axis) shows the difference between integrals at 0.5 and 25 mM Pi, revealing two components that explain the lower force at higher [Pi]: one for *x* > 9 nm (primarily *x* > 12 nm) and one for *x* < 9 nm. The figure is similar to one previously shown by Månsson (2025). **(D)** Integral of ATP turnover rate vs. *x* (left vertical axis) with integration starting at *x* = 15 nm and running in the negative *x*-direction at 0.5 mM (black) and 25 mM (red) Pi. Orange (right vertical axis) shows the difference between 0.5 and 25 mM Pi, revealing only one component of the ATP turnover rate (at *x* < 9 nm) that explains the lower rate at high [Pi]. **(E)** Difference between the force integrals in panel C (orange) superimposed on the difference (blue) in the integrals of *N*_*a*_ vs. *x* at 0.5 and 25 mM Pi. The blue *ΔN*_*a*_ plot reveals just one component (for *x* > 11 nm) of the decrease in *N*_*a*_ with increased [Pi]. The units of *ΔN*_*a*_ (fraction) refer to the fraction of all the myosin heads available along the 36-nm actin filament periodicity.

**FIGURE 5:**
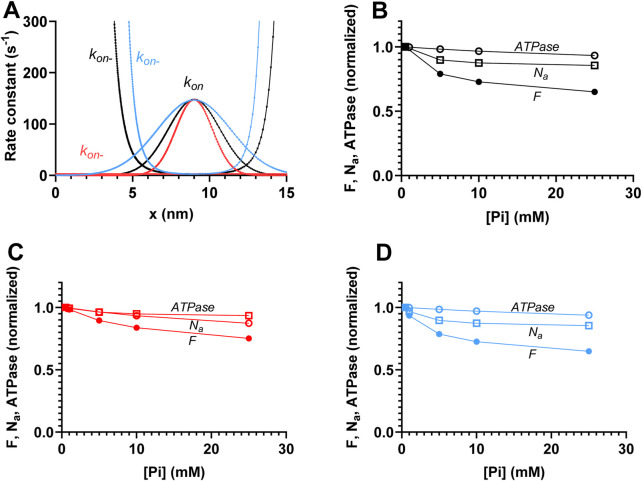
Effects of the width of attachment rate function (*k*_*on*_(*x*)) on the relationships between force (*F*), ATPase activity (*ATPase),*, and the number of attached cross-bridges (*N*_*a*_) during steady-state isometric contraction vs. [Pi]. **(A)** Attachment rate function (*k*_*on*_ *(x)*) and its reversal (*k*_*on-*_(*x*)) are shown using the standard parameter values and the functional forms in the table in Figure 2C with *γ* = 2 (black), *γ* = 1 (red), or *γ* = 4 (blue). All the versions of the rate functions are consistent with the free energy diagrams in Figure 2C so that *ΔG*_*on*_ = -*k*_*B*_ *T* ln (*k*_*on*_(*x*)/*k*_*on-*_(*x*)). Standard deviation for Gaussian functions fitted to *k*_*on*_(*x*) are 1.69 nm (black), 1.195 nm (red), and 2.39 nm (blue). **(B)** *ATPase*, *F*, and *N*_*a*_ vs. [Pi] for the black curves in A. **(C)** *ATPase*, *F*, and *N*_*a*_ vs. [Pi] for the red curves in A. **(D)** *ATPase*, *F*, and *N*_*a*_ vs. [Pi] for the blue curves in A. Data in B to D are normalized to the simulated values at 0.5 mM Pi.

**FIGURE 6:**
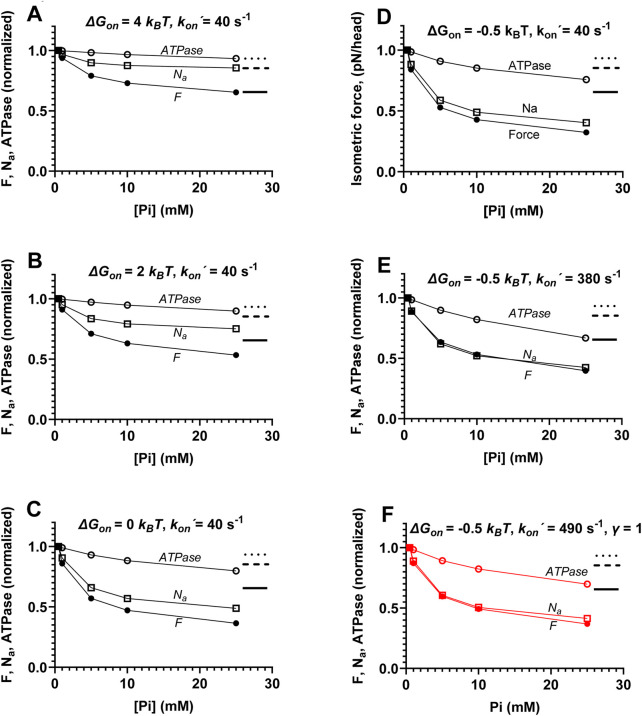
Effects of varied actin affinity in the AMDP state (related to *ΔG*_*on*_) on the [Pi]-dependence of isometric force (*F*), *ATPase*, and *N*_*a*_. **(A)** Standard parameter values are as shown in Supplementary Tables S1, S2, including *ΔG*_*on*_ = 4 *k*_*B*_ *T.* **(B)** As in A, but *ΔG*_*on*_ = 2 *k*_*B*_ *T*. **(C)** As in A, but *ΔG*_*on*_ = 0 *k*_*B*_ *T*. **(D)** As in A, but *ΔG*_*on*_ = −0.5 *k*_*B*_ *T*. **(E)** As in A, but *ΔG*_*on*_ = −0.5 *k*_*B*_ *T* and *k*_*on*_ *´* = 380 s^-1^, making the maximum value of *k*_*on*_(*x*) (*k*_*on*_ (9)) equal to the value with the standard parameter values, as in A. **(F)** As in A, but *ΔG*_*on*_ = −0.5 *k*_*B*_ *T*, *γ* = 1, and *k*_*on*_ *´* = 490 s^-1^, making the maximum value of *k*_*on*_(*x*) (*k*_*on*_ (9)) equal to the value with the standard parameter values. These simulated results are compared to experimental data from the literature in Supplementary Figure S1, where the simulations are also reproduced to mimic (*cf.* Supplementary Table S3) the lower temperature of the experiments. All data are normalized to simulated values at 0.5 mM Pi. The dotted, dashed, and full horizontal lines to the right in each panel indicate the reduction in *ATPase, N*_*a*_, and *F* upon increasing Pi from 0.5 to 25 mM for the conditions in panel A.

**FIGURE 7:**
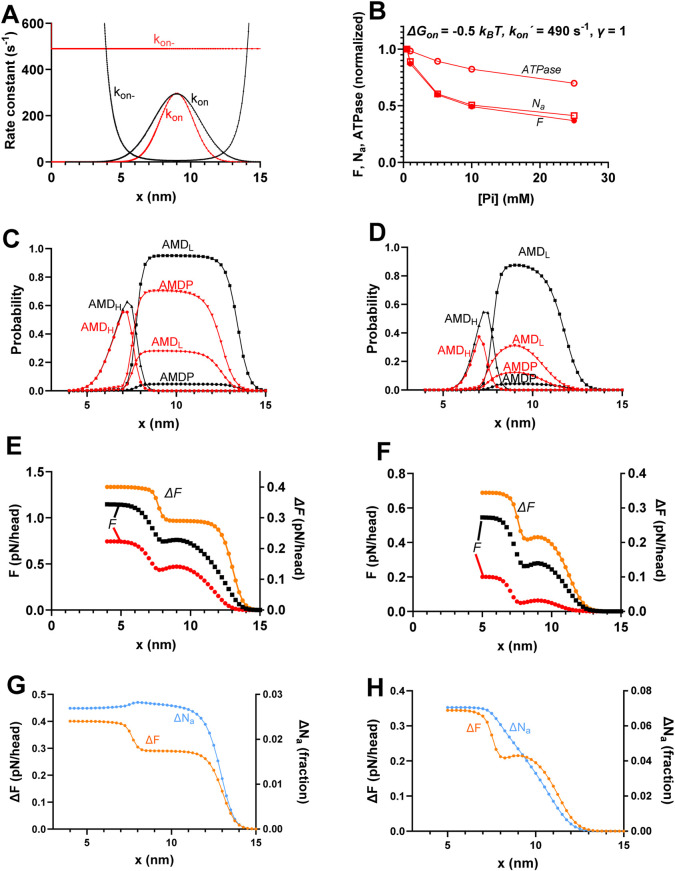
Effects of realistic narrowing of cross-bridge distribution compared to the standard conditions. **(A)** Attachment rate function (*k*_*on*_(*x*)) and its reversal ((*k*_*on-*_(*x*)) using the standard parameter values (*γ* = 2; black) or *γ* = 1, *ΔG*_*on*_ = −0.5 *k*_*B*_ *T*, and *k*_*on*_ *´* = 490 s^-1^ (red; same as in Figure 6F). **(B)** Replotting of data in Figure 6F predicted by the model corresponding to the red curve in A. **(C)** Probability distribution of cross-bridge states (summing up to 1 for each *x* value) during steady-state isometric contraction for standard parameter values for [Pi] = 0.5 mM (black) and 25 mM (red). The data are same as in Figure 4B. **(D)** Probability distribution of cross-bridge states as in C but simulated from the mechanokinetic model for the modified conditions *γ* = 1, *ΔG*_*on*_ = −0.5 *k*_*B*_ *T*, and *k*_*on*_ *´* = 480 s^-1^ for [Pi] = 0.5 mM (black) and 25 mM (red). **(E)** Integral of force (left vertical axis) vs. *x* with integration starting at *x* = 15 nm and running in the negative *x*-direction at 0.5 mM (black) and 25 mM Pi (red). Orange (right vertical axis) shows the difference between 0.5 and 25 mM Pi, revealing two components of force decrease between 0.5 and 25 mM Pi. The data are same as in Figure 4C. **(F)** Same type of data and same color coding as in E but simulated for the condition with *γ* = 1, *ΔG*_*on*_ = −0.5 *k*_*B*_ *T*, and *k*_*on*_ *´* = 480 s^-1^, corresponding to cross-bridge distributions in D with integration starting at x = 15 nm. **(G)** Difference between the force integrals replotted from panel E and Figure 4C (orange) superimposed on the difference (blue) in integrals of *N*_*a*_ vs. *x* at 0.5 and 25 mM Pi replotted form Figure 4E. The *ΔN*_*a*_ plot reveals that the decrease in *N*_*a*_ with increased [Pi] is attributed only to cross-bridges for *x* > 11 nm. **(H)** Same type of data and color coding as in G but simulated for the condition with *γ* = 1, *ΔG*_*on*_ = −0.5 *k*_*B*_ *T*, and *k*_*on*_ *´* = 480 s^-1^. The ΔF plot was reproduced from panel F. The *ΔN*_*a*_ plot reveals that the decrease in *N*_*a*_ with increased Pi is approximately proportional to the decrease in force for all *x*-values.

Below, we use the mechanokinetic model in Figure 2 starting with parameter values (Supplementary Tables S1, S2) previously shown (Månsson, 2025) to provide accurate predictions of force–velocity data, Pi-transients, and other effects of varied [Pi]. The model structure is identical to that in the previous paper (Månsson, 2025), and we re-use some results from that study (Figure 3 and Figures 4B, C) to provide a context for the present work. The key new results are the effects of varied Pi on the number of attached cross-bridges and the ATP turnover rate (remaining data in Figures 4–7 and associated text). The similarity with the previous paper (Månsson, 2025) is summarized in the following points: 1. the methodology is essentially unchanged, 2. the work extends previous simulations by considering the effects of varied [Pi] on the ATP turnover rate (main focus) and on the number of attached cross-bridges, generating predictions for these phenomena, and 3. different parameter values beyond those considered previously are evaluated for their effects on the relationship between [Pi] on one hand and isometric force, isometric ATPase, and the number of attached cross-bridges on the other.

The structure of the model and the free-energy diagrams for each cross-bridge state are shown in Figures 2A, B. The functions defining the strain-dependence of rate constants are shown in Figure 2C, which largely replicates those from Månsson (2025) and are also similar to functions used previously (Eisenberg et al., 1980; Månsson, 2021; Moretto et al., 2022; Månsson and Rassier, 2022). A position coordinate, *x*, quantifies the cross-bridge strain as the distance between the closest actin filament binding site and a myosin head. It is defined such that *x* = 0 nm corresponds to the distance where the myosin head binds in the rigor (AM) state at its minimum free energy. The main force-generating transition in the model in Figure 2 is the AMD_L_ to AMD_H_ transition, which is associated with a swing of the lever-arm during muscle shortening. We will denote this transition as the power-stroke, following the standard use in the biochemical and biophysical literature (reviewed by Geeves, 2016; Robert-Paganin et al., 2020; and Rassier and Månsson, 2025).

The force in each state at a given *x*-value is calculated as the product of the state probability and the gradient, *k*_*s*_ (*x*-x_i_), of the free energy profile. Here, *k*_*s*_ is the cross-bridge stiffness, and *x*_*i*_ (*x*_*1*_, *x*_*2*_, or *x*_*3*_) is the *x*-value, set in the model (*cf.* Figure 2B), where the given state has its minimum free energy. The myofibril or muscle force is obtained by multiplying the cross-sectional area of the preparation with the sum of the forces per cross-sectional area due to all cross-bridge states (averaged over the actin filament periodicity of 36 nm). The population of all the cross-bridge states during steady-state isometric contraction is approximated by the steady-state solution of differential equations in the state probabilities for a very low relative actin-filament gliding velocity (0.7 nm/s). Force *<F>* and the number of attached cross-bridges *<N*_*a*_ *>* averaged over the 36-nm periodicity are derived from the state probabilities:

$$<F>=\frac{\int_{-18}^{18}k_{s}\left(\left(\left[\text{AMDP}\right]\left(x\right)+\left[{\text{AMD}}_{L}\right]\left(x\right)\right)\left(x-x_{1}\right)+\left[{\text{AMD}}_{H}\right]\left(x\right)\left(x-x_{2}\right)+\left[\text{AMD}\right]\left(x\right)\left(x-x_{3}\right)\right)dx}{36},$$ (1)

$$<N_{a}>=\frac{\int_{-18}^{18}\left(\left(\left[\text{AMDP}\right]\left(x\right)+\left[{\text{AMD}}_{L}\right]\left(x\right)\right)+\left[{\text{AMD}}_{H}\right]\left(x\right)+\left[\text{AMD}\right]\left(x\right)\right)dx}{36}.$$ (2)

The averaged ATP turnover rate *< ATPase>* is derived as follows:

$$<ATPase>=\frac{\int_{-18}^{18}k_{off}\left(x\right)\left[\text{AMD}\right]\left(x\right)dx}{36}.$$ (3)

Below, we simplify the terminology such that *F ≡ <F>*, *N*_*a*_ *≡ <N*_*a*_ *>*, and *ATPase ≡ <ATPase>.* Further details of the procedure are described in the Supplementary Material and in Månsson (2025). The differential equations were solved numerically using the Runge–Kutta–Fehlberg (4/5) algorithm, as implemented in the program Simnon (Elmqvist, 1975), which is reproduced in the Supplementary Material (see also Månsson, 2019; Moretto et al., 2022; Månsson and Rassier, 2022; and Månsson, 2025).

## 3 Results

Predictions by the mechanokinetic model in Figure 2 of the force–velocity relationship, Pi-transients, and rate of redevelopment of isometric force at varied [Pi] are depicted in Figure 3. These data are reproduced from our recent modeling study (Månsson, 2025) using optimal parameter values (Supplementary Tables S1, S2).

The model provides a fair reproduction of the force–velocity relationship (Figure 3A), although the maximum power is slightly low, as is common for models of this type (*cf.* Edman et al., 1997; Månsson, 2010; and Marcucci and Reggiani, 2016). The model predicts biphasic Pi-transients (Figure 3B) in contrast to the experimentally observed single exponentials (Dantzig et al., 1992; Tesi et al., 2000; Stehle, 2017). However, the fast exponential component has a small amplitude (<20%), and single exponential fits generate reasonably similar rate constants at varied [Pi] for the Pi-transients and the rate of redevelopment of force from 0 (Figure 3C).

We next used the mechanokinetic model to compare its predictions for the effects of increased [Pi] on isometric force and isometric ATPase activity (Figure 4A). The observed predictions of significantly larger reduction in force than in ATPase are consistent with experimental findings from fast rabbit skeletal muscle (Potma et al., 1995) and earlier work using similar mechanokinetic models (Smith, 2014; Månsson, 2021). Experimental data for force vs. [Pi] show variability between studies (Potma et al., 1995; Tesi et al., 2002; Caremani et al., 2008), while only one full experimental set of isometric ATPase data could be found (Potma et al., 1995). The mentioned experimental results are reproduced in Supplementary Figure S1. The basis for the different effects of increased [Pi] on force and ATPase in mechanokinetic models was not considered in detail previously (Smith, 2014; Månsson, 2021). In Figure 4B, we show that they are explained by the changes in the cross-bridge distributions vs. *x* upon increased [Pi] (Figure 4B). The associated force-integrals at 0.5 and 25 mM Pi and their difference (Figure 4B) show that the reduced force with increased [Pi] is primarily due to loss of highly strained myosin cross-bridges in the pre-power-stroke AMDP- and AMD_L_-states (for (*x*-*x*_*1*_) > ∼1 nm). The lower force at high Pi is only to a minor degree due to loss of cross-bridges in the AMD_H_-state (for *x* ≈ *x*_*1*_). However, only the latter, minor loss of force-producing cross-bridges contributes to the reduced ATP turnover rate, as illustrated by the ATPase integrals at 25 mM and 0.5 mM Pi and their difference (Figure 4C). This is because only the cross-bridges lost from the AMD_H_-state would have completed the entire ATPase cycle. The cross-bridges in the pre-power-stroke states at large (*x*-*x*_*1*_), which are responsible for a major fraction of the force loss, cannot transition to the post-power-stroke states and, therefore, do not contribute to ATP turnover.

In a study on skinned rabbit psoas muscle fibers, Caremani et al. (2008) found that increased [Pi] (up to 25 mM added Pi) reduced the half-sarcomere stiffness during steady-state isometric contraction almost to the same degree as the steady-state tension. After correction for the effects of series-compliant elements, the results suggest that the reduction in tension is directly proportional to a reduction in the number of attached cross-bridges. This interpretation was found to be consistent with a kinetic scheme, such as that shown in Figure 1, if the relative contribution to both force and stiffness is identical for both attached cross-bridge states. In contrast to the experimental findings of Caremani et al. (2008) and their interpretation in terms of the kinetic scheme, Smith (2014) found that mechanokinetic models similar to that used here (i.e., with Pi-release before the power-stroke) predict a smaller reduction in the number of attached cross-bridges (*N*_*a*_) than in isometric force with increased [Pi]. It was of interest to test whether the present model provides similar predictions. Figure 4A shows that it does. That is, the reduction in *N*_*a*_ for an increase of [Pi] from 0.5 to 25 mM is less than half of the reduction in isometric force. The cross-bridge distributions and the force and *N*_*a*_-integrals in Figures 4B, C, E reveal the mechanism behind the difference. It follows from the integrals that the decrease in force has two components, whereas the decrease in *N*_*a*_ only has one (Figure 4E). The decrease in force with an increase in [Pi] is primarily due to a loss of cross-bridges in the AMDP and AMD_L_ states at large *x* (*x* > *x*_*1*_ *)*. The biggest decrease occurs for *x* > 12 nm (orange line in Figure 4C). In addition, a component of the decrease is attributed to the reversal of the power-stroke (AMD_H_ → AMD_L_ transition) for *x* ≈ *x*_*1*_. The latter effect is, in turn, a mass-action effect due to a shift of the equilibrium from the AMD_L_ to the AMDP state upon increased [Pi]. The decrease in *N*_*a*_ with increasing [Pi], on the other hand, is solely attributable to the loss of AMDP and AMD_L_ cross-bridges for *x* > *x*_*1*_ (primarily *x* > 12 nm; Figure 4E). The equilibrium-shift toward the AMDP state for *x* ≈ *x*_*1*_ does not result in a decrease in *N*_*a*_ due to a high binding affinity for the AMDP state at that *x*-value (*cf.* free energy diagrams in Figure 2B).

Attachment of cross-bridges in the AMDP and AMD_L_ states for large values of *x* is central for the dominant component of force decrease with increased [Pi] and also for the different effects of increased [Pi] on isometric force and ATPase. The behavior is consistent with the non-negligible actin affinity in the AMDP states at *x* > 11 nm, as observed from the free energy diagrams (Figure 2B) with the rate functions *k*_*on*_ *(x)* and *k*_*on-*_ *(x)*, obeying the relationship *ΔG*_*on*_(*x*) = -*k*_*B*_ *T* ln (*k*_*on*_ (*x*)/*k*_*on-*_(*x*)). We assume, in accordance with earlier implementations (Eisenberg et al., 1980; Månsson, 2021; Moretto et al., 2022; Månsson and Rassier, 2022), a Gaussian attachment rate function and a detachment rate function that is equal to the inverted attachment function (black curves in Figure 5A). The Gaussian attachment rate function in this case has a larger standard deviation $SD=\sqrt{<x^{2}>}$ than expected from the thermal motion of the myosin heads according to the equipartition theorem, where $<x^{2}>$ = *k*_*B*_ *T/k*_*s*_. With the numerical values *k*_*B*_ *T* = 4 pN nm and *k*_*s*_ = 2.8 pN/nm, the equipartition theorem would result in SD ≈ 1.195 nm (red in Figure 5A) instead of 1.69 nm, which characterizes the standard attachment rate function (black) in Figure 5A. This is consistent with a dual-attachment mechanism that broadens the attachment rate function compared to that predicted by thermal motion only (Smith et al., 2008). Such a mechanism has been widely assumed since the work of Eisenberg et al. (1980). It was, however, be of interest to investigate whether reducing the width of the *k*_*on*_(*x*)-function to 1.195 nm would bring the behavior closer to that of a kinetic scheme as this is expected to produce a narrower cross-bridge distribution. To implement this possibility, we changed *k*_*on*_(*x*) and *k*_*on-*_(*x*), as shown by the red lines in Figure 5A, with *k*_*on-*_(*x*) now being constant, independent of *x*, and consistent with the free energy diagrams in Figure 2B. We also tested the opposite change with the broadened attachment rate function (blue in Figure 5A) and changed *k*_*on-*_(*x*) for consistency with the free energy diagram. The effects of the different functions *k*_*on*_(*x*) and *k*_*on-*_ *(x)* in Figure 5A on the [Pi]-dependence of isometric force, isometric *N*_*a*_, and isometric ATPase (calculated from Equations 1–3) are depicted in Figures 5B–D. Narrowing of *k*_*on*_(*x*) (reduced standard deviation down to 1.195) with the constant value of *k*_*on-*_(*x*) reduced the effect of Pi on both force and *N*_*a*_ but slightly increased the Pi-induced reduction in *ATPase* (Figure 5C) compared to the standard conditions (Figure 5B). However, the magnitude of the effect on force was still higher than that on both *ATPase* and *N*_*a*_ *,* as for the standard model parameters. Increasing the standard deviation of *k*_*on*_(*x*) to 2.39 nm by increasing *γ* to 4 negligibly modified the Pi-dependence of force, *N*_*a*_, and *ATPase* (Figure 5D) compared to the standard parameter values shown in Figure 5B.

Another way to reduce the width of the cross-bridge distribution would be to reduce the actin-affinity in the AMDP state by reducing *ΔG*_*on*_. Such a parameter change is also of interest to test because a previous model study (Smith, 2014) suggested that it would affect the Pi-sensitivity of force. Lowering *ΔG*_*on*_ from 4 to −0.5 *k*_*B*_ *T* (Figures 6A–D) increases the Pi-sensitivity of force, which is consistent with previous results (Smith, 2014). It also increases the Pi-sensitivity of the isometric ATPase but maintains an appreciably larger effect of increased [Pi] on force than on ATPase. Finally, interestingly, the reduction in *ΔG*_*on*_ had the greatest relative effect on number of attached cross-bridges, making the decrease in *N*_*a*_ with increased [Pi] almost proportional to the decrease in force. Full proportionality was obtained (Figure 6E) if the maximum value of *k*_*on*_(*x*) was unchanged by increasing *k*_*on*_ *´* from 40 s^-1^ to 380 s^-1^ to compensate for the decrease in the exponential factor in *k*_*on*_(*x*) (Figure 2C). Clearly, this set of parameter values is the one tested, which accounts best for the effects of varied Pi on force, ATPase, and the number of attached cross-bridges. However, it will result in greater deviation of Pi-transients from a single-exponential function due to the larger amplitude of the fast component of the transient. This reflects the difficulty of identifying a single model and set of parameter values that can account for all the effects of varied [Pi] (and all other contractile properties). However, importantly, the main aim of the present study is more modest than that, as discussed further below.

Finally, we combined a narrowed attachment rate function (*k*_*on*_(*x*), achieved by reducing *γ* from 2 to 1) with lowered *ΔG*_*on*_ from 4 to −0.5 *k*_*B*_ *T* to obtain as narrow a cross-bridge distribution as possible within a realistic range. These combined changes (with *k*_*on*_ *´* increased to 490 s^-1^) resulted in closely similar Pi-dependencies (Figure 6F) of isometric force, *ATPase*, and *N*_*a*_ as the same changes in *ΔG*_*on*_ with *γ* = 2 (Figure 6E). In Figure 7, we explore the mechanisms underlying these effects. The rate functions *k*_*on*_ *(x)* and *k*_*on-*_ *(x)* with (*γ* = 1, *ΔG*_*on*_ = −0.5 *k*_*B*_ *T*, and *k*_*on*_´ = 490 s^-1^) and without (*γ* = 2, *ΔG*_*on*_ = 4 *k*_*B*_ *T*, *and k*_*on*_´ = 40 s^-1^) the changes are given in Figure 7A. It is clear from the analysis (Figures 7B–F) that the larger decrease in force with increased [Pi] for *ΔG*_*on*_ = −0.5 *k*_*B*_ *T* and *γ* = 1 than that under standard conditions (see Figure 4) is due to the greater loss of both the cross-bridges from the AMDP and AMD_L_ states at large *x* and from the AMD_H,_ AMDP, and AMD_L_ states at *x* close to *x*_*1*_. The loss of cross-bridges at large *x* with increased [Pi] still dominates the force-effect, as observed from the difference between the force integrals in Figure 7F (orange curve). This explains why the effect of increased [Pi] on ATPase is smaller than that on force with the narrow cross-bridge distribution because only the smaller component of loss in force is associated with reduced ATPase. The results in Figures 7G, H, where the differences in the force integrals and *N*_*a*_-integrals between 0.5 mM and 25 mM Pi are superimposed, illustrate why reduced actin affinity leads to a proportional decrease in both force and *N*_*a*_. In contrast to the standard conditions (Figure 7G; same data as in Figure 4), cross-bridges with low actin-affinity (*ΔG*_*on*_ = −0.5 *k*_*B*_ *T* and *γ* = 1; Figure 7H) detach to the MDP state for almost all *x*-values that contribute to a decrease in force upon increased [Pi].

Detachment at *x* ≈ *x*_*1*_ is not observed when the actin affinity is as high as under our standard conditions (Figure 7G). However, under the latter conditions, cross-bridges in this *x*-range contribute to the decrease in force with increased Pi through the shifted equilibrium from the high-force AMD_H_ state over the low-force AMD_L_ to the likewise low-force AMDP state.

## 4 Discussion

### 4.1 Summary of the results and comparison to earlier mechanokinetic models

The present study starts with a recent mechanokinetic model (Månsson, 2025) (Figure 2) that provides reasonable predictions of the force–velocity relationship and the effects of varied Pi on Pi-transients, steady-state isometric force, and the rate of force redevelopment from 0 (Figure 3). Using this model with the optimal parameter values from the previous study (Figure 2; Supplementary Tables S1, S2), we first corroborated earlier results (Smith, 2014; Månsson, 2021) on the energetics of steady-state isometric contraction. Particularly, we show that the model in Figure 2 predicts an appreciably smaller effect of increased [Pi] on the ATP turnover rate than on force during steady-state isometric contraction of fast skeletal muscle fibers (Figure 4A). We also show that this follows in the model because the effect of increased [Pi] on isometric force is primarily due to a component attributed to highly strained pre-power-stroke cross-bridges that do not go through complete ATP turnover cycles (Figures 4B–D). Thus, the lower effect of increased [Pi] on ATPase than on force is an inherent feature of the mechanokinetic model. In contrast, for the simple kinetic scheme (Figure 1) (Kawai and Halvorson, 1991; Dantzig et al., 1992; Ranatunga, 1999; Tesi et al., 2000; Stehle, 2017; Kawai et al., 2021; Stehle, 2024), where it was necessary to add a branched pathway (Linari et al., 2010) to account for the different effects on ATPase and force.

We recently optimized the parameter values of the mechanokinetic model to those (Månsson, 2025) in Supplementary Tables S1, S2. In that process, we changed the values of x_1_ and x_2_ to modulate the steady-state populations of the AMDP, AMD_L_, and AMD_H_-states during isometric contraction to minimize a fast component of the biphasic Pi-transients (Figure 3B) while maintaining a reasonable force–velocity relationship (Figure 3A). Thus, the amplitude of the second sub-stroke AMD_H_-> AMD (x_2_–x_3_) relative to the major sub-stroke AMD_L_-> AMD_H_ (x_1_–x_2_) affects both steady-state shortening and the fraction of force-producing cross-bridges during isometric contraction that complete ATP turnover cycles. Evidence for a second sub-stroke has been provided by different types of studies (Whittaker et al., 1995; Veigel et al., 2003; Capitanio et al., 2006; and Albet-Torres et al., 2009; reviewed by Rassier and Månsson, 2025), and this sub-stroke is important for conferring strain-sensitivity to cross-bridge detachment from the AMD_H_ state (e.g., Duke, 1999; Nyitrai and Geeves, 2004; and Albet-Torres et al., 2009). This ensures different cycling kinetics between isometric contraction and shortening, and, of more specific relevance to this work, it modulates the contribution of the AMD_H_-state (and thereby the “cycling component”) to steady-state isometric force.

It was previously found that the kinetic scheme in Figure 1 is consistent with a proportional reduction of isometric force and the *N_a_* (Caremani et al., 2008) on the assumption that all attached cross-bridge states contribute equally to force (Caremani et al., 2008). We show here (Figures 4A, 5) that our mechanokinetic model with the optimized parameter values from the previous study (Månsson, 2025) does not reproduce this result. However, similar to the kinetic scheme of Caremani et al. (2008), we find parameter values for the mechanokinetic model that lead to the proportional reduction of force and *N_a_* with increased [Pi], while maintaining a smaller effect on ATPase (Figure 6).

The different effects of varied [Pi] with varying parameter values are interesting (see also Smith, 2014; Moretto et al., 2022) in relation to quite substantial experimental differences between muscle types (Potma et al., 1995; Tesi et al., 2002) and at varied temperatures in a given muscle type (Coupland et al., 2001). Further exploration is, however, outside the scope of this study because an appreciably wider range of parameter values would need to be considered to fully elucidate the effects.

### 4.2 Detailed comparison of mechanokinetic models and simple kinetic scheme

The use of the simple kinetic scheme in Figure 1 to explain the effects of varied [Pi] in terms of the Pi-release-rebinding process is natural considering its simplicity combined with the wide range of experimental results accounted for (reviewed by Kawai, 2025). However, the simplicity of the scheme may be deceptive if all essential features of the experimental system are not considered. We have partly discussed this issue before (Månsson, 2025), highlighting the difficulty in integrating a separate force-generating transition (with myosin lever arm swing) into the scheme with maintained predictive capacity. A model without such characteristics is expected to have a low maximum power output. A possibility to circumvent the latter problem was proposed by Caremani et al. (2008). To that end, they suggested that both force-generating states (AMDP and AMD) in Figure 1 exist in one pre-power-stroke and one post-power-stroke conformation in rapid equilibrium with each other with the same equilibrium constant, whether Pi is in the active site or not. This necessary constraining condition compromises the simplicity. The simplicity is further compromised by the need to add a branched pathway to account for different effects on isometric force and isometric ATPase. Therefore, the present mechanokinetic model may be viewed as a simpler alternative. It has a natural force-generating transition with a lever arm swing (AMD_L_ - > AMD_H_) that does not severely compromise the capability to account for Pi-transients (Smith, 2014; Månsson, 2025), and it naturally accounts for different effects of varied [Pi] on isometric force and ATPase. We also argue that isometric contraction involves such a wide range of elastic strains of each attached cross-bridge state that it is not durable to use a simple kinetic scheme to interpret the experimental results. This follows from our analysis in Figures 5–7, where we varied model parameter values within experimentally reasonable ranges with the aim of obtaining narrower cross-bridge distributions. If the latter become sufficiently narrow, one would expect (Linari et al., 2010) that the model could be well-approximated by a simple kinetic scheme. If that had been the case, one would expect to lose the ATP-insensitive component due to cross-bridges in the AMD_L_ and AMDP states for *x* > *x*_*1*_ and, therefore, also lose the difference between the Pi-sensitivity of isometric force and isometric *ATPase*. However, our analysis does not lend support to that idea. While the [Pi]-sensitivity of ATPase increased for the case with a narrower cross-bridge distribution, the Pi-sensitivity of force increased further (*cf.* Smith, 2014), thereby maintaining the different Pi-sensitivity of force and ATPase. In further support of the mechanokinetic model, we also found that the larger Pi-induced decrease in force with a narrower cross-bridge distribution is accompanied by a proportional reduction of the number of attached cross-bridges, consistent with the experimental results (Caremani et al., 2008).

Table 1 summarizes the most central aspects of the differences and similarities between the mechanokinetic model in Figure 2 and the kinetic scheme in Figure 1, with emphasis on the capabilities to predict the important effects of varied [Pi].

**TABLE 1:** Comparing the kinetic scheme in Figure 1 with the mechanokinetic model in Figure 2.

Property, predictive capability	Kinetic scheme	Mechanokinetic model
Can account for interventions including length changes, e.g., force–velocity data	**No**	**Yes**
Predicts single-exponential Pi-transients with k_Pi_ = k_tr_ and similar saturating Pi-dependence	**Yes**	**Approximately** (see Månsson, 2025)
Predicts similar Pi-sensitivity of force and *N*_*a*_	**Yes**	**Yes**
Naturally (without adding branched pathways) predicts lower Pi-sensitivity of isometric ATPase than of isometric force	**No**	**Yes**
Number of components contributing to force	**One**	**Two**, one with and one without power-stroke. Potential physiological importance

### 4.3 Two components of isometric force: properties and implications for muscle physiology

The existence of different components of isometric force is a natural consequence of models featuring more than one attached state, a power-stroke between them, and a distribution of cross-bridge strains. The present analysis (see also Månsson, 2025) explicitly demonstrates the existence of two distinct components and their differing properties. Out of the two components, one is due to cross-bridges that hold force already upon attachment (AMDP and AMD_L_ state for *x* > *x*_*1*_). This represents a Brownian ratchet mechanism that depends on thermal fluctuations captured by an asymmetric potential, as in the original (Huxley, 1957) model. We denote this as the “non-cycling component.” The other component of isometric force depends on a lever arm swing (Huxley and Simmons, 1971) or tensing of an elastic element associated with the lever arm (Eisenberg and Hill, 1978). We denote this as the “cycling component” of isometric force because the cross-bridges may release ADP, followed by rebinding of ATP to complete a full ATP turnover cycle. We discuss the properties of these components in greater detail and consider their possible physiological roles.

The non-cycling component, which is maximal in amplitude for *x* ≈ 13 nm (1.4 *x*_*1*_; maximum slope of integral at 0.5 mM Pi in Figure 4C), is associated with slow cross-bridge attachment due to the Gaussian function *k*_*on*_(*x*) centered at *x* = *x*_*1*_. This means that while the non-cycling component is dominant in isometric contraction, its fractional contribution to tension will decrease with increasing shortening velocity. First, this means that it will not contribute significantly to the maximal power output that occurs for shortening at a velocity of several thousand nm/s in fast skeletal muscle (Figure 3A). However, a large fractional contribution during isometric contraction contributes significantly to a high maximum isometric force without contributing to the ATP consumption. Moreover, this energetically “cheap” component is expected to contribute to stabilizing the sarcomere length at tension levels close to the maximum isometric force. This would be achieved by reducing the maximum force in half-sarcomeres that shorten by stretching other weaker half-sarcomeres (Edman and Reggiani, 1984). The decrease in force in the shortening half-sarcomeres is due to the loss of the strongest of the non-cycling cross-bridges because of the slow attachment rate. This is consistent with characteristic features (Edman et al., 1997; Månsson, 2010) of a non-hyperbolic force–velocity relationship found in a wide range of skeletal muscle preparations (Edman, 1988; Edman et al., 1997; Edman, 2005; Devrome and MacIntosh, 2007), including whole mammalian muscle *in situ* (Devrome and MacIntosh, 2007). A similar decrease in force as in strong half-sarcomeres due to the reduced attachment of cross-bridges would not be expected to occur in the overall weaker, thus elongating, half-sarcomeres. Although not explicitly studied here, this may be inferred from different and incompletely understood mechanokinetic mechanisms during the elongation of the active muscle (Lombardi and Piazzesi, 1990; Mansson, 1994; Brunello et al., 2007; Nocella et al., 2013; reviewed in Rassier and Månsson, 2025).

The contribution of the cycling component to isometric force is smaller or even markedly smaller than that of the non-cycling component. The cycling component would, however, dominate during shortening with completed lever arm swings and would be essential to achieve the high maximum power output of the muscle. Most likely, its role in isometric contraction, explaining that it is not completely absent, is to provide preparedness for rapid changes, e.g., in load. Specifically, it would contribute to a resistance to stretch over a larger range that would not be possible if only the non-cycling component with cross-bridges in the AMDP and AMD_L_ states had been present during isometric contraction (Lombardi and Piazzesi, 1990; Mansson, 1994). The cycling component may also assist rapid relaxation that relies on the development of inter-sarcomere non-uniformity (Edman and Flitney, 1982).

### 4.4 General considerations for modeling of Pi-release and force-generation

A wide range of models has been proposed to account for the temporal relationship between Pi-release from the active site and force-generation (reviewed by Debold, 2021; Månsson et al., 2023; and Rassier and Månsson, 2025). Most models assume Pi-release either strictly before or strictly after the power-stroke; few models assume concomitant Pi-release and power-stroke (Pate and Cooke, 1989). Finally, there are models assuming “loose coupling” between Pi-release and force-generation, i.e., Pi is released from the active site either before or after the power-stroke, depending on the conditions (Malnasi-Csizmadia and Kovacs, 2010; Caremani et al., 2013; Rohde et al., 2017; Governali et al., 2020). Models with Pi-release after force-generation have also been combined with branched pathways, as considered above for the modification of the kinetic scheme in Figure 1 (Linari et al., 2010). Another model with Pi-release after the power-stroke and a branched pathway assumes Pi-binding to a post-power-stroke state that leads to cross-bridge detachment rather than to a reversal of the power-stroke. This model has been used to explain both the combined effects of varied [Pi] and pH on *in vitro* motility assay results (Debold et al., 2011) and single-molecule and small-ensemble mechanical data of wild-type and mutated myosins (Debold et al., 2013; Scott et al., 2021; Marang et al., 2023; Marang et al., 2025).

The goal of the present study is not to rule out any of the wide range of models briefly considered above. Rather, we wish to expose the simple facts that 1. generally, a mechanokinetic model may generate results that appear to be unexpected and possibly simpler than those of a similar kinetic scheme (see also Månsson, 2025), and 2. specifically, a mechanokinetic model can quite naturally explain the different effects of varied [Pi] on isometric force, *N_a_*, and ATPase. As a basis for our modeling, we use a relatively simple mechanokinetic model within the class that assumes Pi-release from the active site before the force-generating structural change, without any branching. A model of this type accounts for a range of experimental findings (Månsson, 2021; Moretto et al., 2022; Månsson, 2025) and is readily combined with the idea of Pi-binding to secondary sites outside the active site (Moretto et al., 2022; Månsson, 2025) to account for additional experimental results. For models to be valid, it is important that the actual Pi-release and rebinding steps at the active site are fast and that another step, such as cross-bridge attachment or Pi-dissociation from a secondary Pi-binding site on myosin, is rate-limiting (Smith, 2014; Stehle, 2017; Moretto et al., 2022). Otherwise, the model would not be compatible with the high shortening velocity of muscle. This is fulfilled for both the kinetic scheme (Figure 1) and the mechanokinetic model (Figure 2) considered here. We expect that the two components of isometric force with different contributions to stiffness and ATPase would be present in any mechanokinetic model, independent of the temporal order, between Pi-release and force-generation. However, we have not tested this possibility but recognize that there are limitations to all the models considered above, including our models (*cf.* reviews by Stehle and Tesi, 2017; Debold, 2021; Månsson et al., 2023; Kawai, 2025; Kaya, 2025; and Rassier and Månsson, 2025).

### 4.5 Limitations

In line with our modest aims, we do not consider the effects of varying activation, instead assuming full activation of both the thin and thick filaments (Brunello and Fusi, 2024), unlike in some other models (Campbell et al., 2018; Mijailovich et al., 2021). Moreover, consistent with the challenges in modeling mentioned in the previous section (4.4.), we do not perform any detailed broad tests of the predictive power of our model. Naturally, there is a wide range of higher-order complexities that we do not consider. This can be exemplified by those related to whether myosin heads exhibit linear or non-linear elastic properties (Kaya and Higuchi, 2010; Kaya et al., 2017; Månsson et al., 2019; Linari et al., 2020) or if the stiffness varies between cross-bridge states (Kaya and Higuchi, 2010; Wang et al., 2020).

## 5 Conclusion

We found that a mechanokinetic model can account for some combined experimental results more straightforwardly than apparently simpler kinetic schemes. This capability is related to the cycling and non-cycling components of isometric force, as defined above. In addition to their potential physiological importance, these components specifically explain the combined effects of varied [Pi] on isometric force and ATP turnover rate in the mechanokinetic model. They are also consistent with the associated changes in the number of attached cross-bridges in such a model. In addition to the good predictive power, theoretical considerations based on expected thermal fluctuations argue for the use of mechanokinetic models in relating experimental data obtained during isometric contraction to molecular mechanisms. However, with regard to different models for the temporal relationship between Pi-release and force-generation, we remain open to alternative approaches, acknowledging that each is associated with certain uncertainties and limitations. Finally, in the course of this work, it was highly challenging to find a suitable set of experimental muscle data at varied [Pi] for force, *N_a_*, and ATPase. More studies of this type that probe a range of parameters under one given experimental condition would be important to allow critical tests of models such as those considered here.

## Data Availability

The original contributions presented in the study are included in the article/Supplementary Material; further inquiries can be directed to the corresponding author.
