# Supplementary material for: A mechanokinetic actomyosin model predicts different orthophosphate sensitivities of force and ATP turnover rate during isometric muscle contraction
Source: Front Physiol. 2025 Oct 10;16:1659772. doi: 10.3389/fphys.2025.1659772 (PMC12549700; doi:10.3389/fphys.2025.1659772)
Supplement: Supplementary file 1 [file Supplementaryfile1.docx]

Supplementary Information

**Mechanokinetic actomyosin model predicts different [orthophosphate]-sensitivity of force and ATP turnover rate during isometric muscle contraction**

by

Alf Månsson

**Supplementary Materials and Methods**

*Mechanokinetic model - general*

The description below of the mechanokinetic model and its implementation is based on that in a recent paper (Månsson, 2025a) and only minimally modified from that description. Despite the minimal difference, we decided to add the information here to aid readers to get a full grip of the main paper without having to consult references.

**Table S1. Standard parameter values^a^ determining shape of free energy diagrams for simulation of contractile properties of fast mammalian skeletal muscle using mechanokinetic model**

| **Parameter** | **Numerical value** |
| --- | --- |
| *x-values for positions of free energy minima of different states* | |
| *x_1_* (AMDP_,_ AMD_L_) | 9 nm |
| *x_2_* (AMD_H_) | 1.5 nm |
| *x_3_* (AM, AMD) | 0 nm |
| *Differences in free energies between neighboring states* | |
| *******G_on_* (MDP-AMDP) | 4 *k_B_T* (varied as explained in text and figure legends) |
| *******G_P_* (AMDP – AMD_L_) | -*k_B_T* ln([Pi]/*Kp*) |
| *******G_LH_* (AMD_L_- AMD_H_) | 13 k_B_T |
| *******G_HR_* (AMD_H_ –AMD/AM) | 2 k_B_T |
| *$\Delta G_{ATP}$* | 13.1 + ln ([MgATP]/ ([MgADP][Pi]) *k_B_T* (28 k_B_T) |
| *Cross-bridge stiffness* | |
| *k_s_* | 2.8 pN/nm |

Footnotes to Table S1

^a^ The parameter values are estimated primarily from two-headed myosin motor fragments from fast skeletal muscle of the rabbit at 25-30^o^C (see references in text for details).

**Table S2. Standard parameter values^a^ defining rate functions and kinetic constants for simulation of contractile properties of fast mammalian muscle using mechanokinetic model**

| **Parameter** | **Numerical value** |
| --- | --- |
| *k_+3_ + k_-3_*  (Recovery stroke+hydrolysis) | 220 s^-1^ |
| *K_3_* | 10 |
| k_on_´ | 40 s^-1^ (varied as explained in text and legends) |
| *γ* | 2 (varied as explained in text and legends) |
| *k_P+_´* | 10,000 s^-1^ |
| *k_LH-_* | 6,000 s^-1^ |
| *k_5_´* | 2,000 s^-1^ |
| *K_P_* | 10 mM |
| *x_crit_* | 0.6 nm |
| *k_6_* | 5000 s^-1^ |
| Physiological [Pi] | 0.5 mM |
| *K_1_* | 1.7 mM^-1^ |
| *k_2_´* | 2,000 s^-1^ |

Footnotes to Table S2

^a^ The parameter values are primarily from two-headed myosin motor fragments from fast skeletal muscle of rabbit at 25-30^o^C (see references in text for details).

In the mechanokinetic model (main Fig. 2) we use parameter values for fast skeletal muscle at 25 – 30^o^C (Tables S1-S2; (Månsson, 2025a)) based on values that we arrived at previously (Månsson, 2016; 2020; 2021; Moretto et al., 2022). The parameter values are similar to the standard values used in our recent paper except for change of x_1_ from 7.7 nm to 9 nm, x_2_ from 2 to 1.5 nm and k_on_´ from 20 to 40 s^-1^. These changes improved the reproduction of single exponential Pi-transients while maintaining satisfactory predictions of other experimental data. We assume a Hookean cross-bridge elasticity and a uniform x-distribution of the myosin heads relative to the myosin binding sites, separated by 36 nm along the actin filaments.

Steady state isometric cross-bridge distributions with respect to the variable x of the states in Fig. 2A were approximated by solution of differential equations in the state probabilities at varied *x* (Eqs. 1- 6) for very low velocity (v≤0.7 nm/s < 0.007 % of maximum shortening velocity).

$\frac{d[MT]}{dx}$*=(-k_+3_* [MT]*+k_-3_*[MDP]*+k_off_* [AMD]*)/v (1)*

$\frac{d[MDP]}{dx}$*=(k_+3_*[MT]+*k_on-_(x)*[AMDP]-(*k_on_(x)+k_-3_*)[MDP]*)/v (2)*

$\frac{d[AMDP]}{dx}$*=(k_on_(x)*[MDP]+(*k_P_*_+_[Pi]/*K_P_*)[AMD_L_]-(*k_on-_(x)+k_P+_)* [AMDP]*)/v (3)*

$\frac{d[{AMD}_{L}]}{dx}$*=(k_LH-_* $[\mathrm{AMD}_{H}]$ *+ k_P+_*[AMDP]*-( k_LH+_(x)+ k_P+_*[Pi]*/K_P_)* $[\mathrm{AMD}_{L}]$)/*v (4)*

$\frac{d[{AMD}_{H}]}{dx}$*=(k_LH+_(x)*$[\mathrm{AMD}_{L}]$ *-(k_LH-_+k_5_(x))*$[\mathrm{AMD}_{L}$]*)/v (5)*

$\frac{d[AMD]}{dx}$*=(k_5_(x)* $[{A\mathrm{MD}}_{L}$]*-k_off_(x)*[AMD])/*v (6)*

The state probability (indicated by []) is a function of x but we omit the argument (x) for clarity. The numerical computations started at 16 nm and ran in the negative x-direction to cover all x-values with the state probabilities differing from their initial values at high x. The latter initial values were [AMDP]=[AMD_L_]=[AMD_H_]=[AM]*=*0 whereas [MT]=0.1 and [MDP]=0.9 with K_3_=10 (Table 2). To avoid instabilities in the computations, values of rate functions larger than r_max_= 300 000 s^-1^ or smaller than r_min_= 0.001 s^-1^ were set to either of these values or 0 (for r_min_).

*Implementation of the model*

The numerical solutions of the system of non-linear ordinary differential equations were obtained in the program Simnon. Unfortunately, this program is no longer available for purchase. It was originally developed by the Department of Automatic control at Lund University (Elmqvist, 1975) and then, for a period, made commercially available by SSPA Maritime Consulting AB Gothenburg, Sweden. The performance of the system was tested extensively during the development phase and when commercially available. We have previously compared its performance against analytical solutions of differential equations in simple cases (Månsson, 2025b) and against Monte-Carlo simulations implemented in Matlab for more complex models than used here (Månsson and Rassier, 2022). The fact that Simnon is no longer available means that the code cannot be used directly by a commercially available program. However, the extensively commented code given below is readily transformed into other programming languages including Matlab.

CODE:

" comment

--------------------------------------------------------------------------------------

**continuous system mkinmod1**

*"Declaration of program start above*

*"Declaration of states below*

state a00 a0 a11 a1 a2 a3

*"a00:MT; a0:MDP;*

*"a1:AMDP a11:AMDL a2:AMDH; a3:AMD*

state i atpas Na

*"i: derivative of total force*

*"atpas: derivative of ATPase vs x. Na: derivative of Na vs x*

*"Declaration of derivatives below*

der da00 da0 da11 da1 da2

der da3 di datpas dNa

*"Definition of integration variable*

time t

*"Definition of position variable in terms of t*

x=15-t

*"Differential equations d[state]/dx*

da00=(-k3*a00+k3m*a0+koff*a3)/v

da0=(k3*a00+konm*a1-(kon+k3m)*a0)/v

da1=(kon*a0+kpp*(Pi/Kp)*a11-(konm+kpp)*a1)/v

da11=(klhm*a2+kpp*a1-(klh+kpp*(Pi/Kp))*a11)/v

da2=(klh*a11-(klhm+k5)*a2)/v

da3=(k5*a2-koff*a3)/v

*"Differential equations to solve integrals in average Na, force and ATPase*

dNa=(1/36)*(a1+a11+a2+a3)

di=(k/36)*(a2*(x-x2)+(a1+a11)*(x-x1))+(k/36)*a3*(x-x3)

datpas=(1/36)*koff*a3

*"Setting velocity*

v:0.7

*"Initial values for x=14 nm or x=15nm where integration starts*

a00:0.1

a0:0.9

a1:0

a2:0

a3:0

a11:0

*"constants rmax*

fc:300000

fc1:300000

*"constant to limit calculations to certain x-range for stability*

xlimit:3

*"attachment transitions between a0 and a1 (MDP and AMDP)*

kon1=kon0*exp((1/gamma)*(Gon-(k/2)*(x-(x1))*(x-(x1))/4))

*"Division by 4 here and below because 1kBT=4 pN nm with k in units of "pN/nm*

kon=if x>(x1+3*xlimit) then 0 else if x<(x1-3*xlimit) then 0 else kon1

konm1=kon0*exp((1-1/gamma)*(-Gon+(k/2)*(x-x1)*(x-x1)/4))

konm11=if (konm1>fc1) then fc1 else if x<(x1-3*xlimit) then fc1 else konm1

konm=if x<(x1+3*xlimit) then konm11 else fc1

*"transitions between a11 and a2 (Huxley-Simmons transition;AMDL "->AMDH)*

exp_12=(k/2)*(x-x1)*(x-x1)/4-(k/2)*(x-x2)*(x-x2)/4

klh1=klhm*exp(GLH+exp_12)

klh=if (klh1>fc1) then fc1 else klh1

*"Transition from a2 to a3 (AMDH -> AMD)*

exp_2=(k/2)*(x-x2)*(x-x2)/4-(k/2)*(x-x3)*(x-x3)/4

k51=if k50*exp(G23+exp_2)>fc1 then fc1 else k50*exp(G23+exp_2)

k5=if (k51>fc) then fc else k51

*"Parameters for Bell-detachment rates below*

fb=exp(k*abs(x-x3)*xc/4)

koff0=fb*k20*ATP/((1/(K1))+ATP+(k20*fb/k6)*ATP)

koff=if x<(x1+4*xlimit) then koff0 else 0

*"DECLARATION OF PARAMETER VALUES BELOW. These values are easily modified from command window when running program*

*"Rate constants (s-1*)

k3:200

k3m:20

kon0:40

klhm:6000

k50:2000

k20:2000

*"Rate constant of Pi dissociation*

kpp:10000

Kp:10

K1:1.7

k6:5000

*"Substrate concentration (mM)*

ATP:5

*"Pi concentration (mM)*

Pi:0.5

*"critical detachment distance*

xc:0.6

*"γ*

gamma:2

*"FREE ENERGY DIAGRAMS BELOW*

*"Differences in free energy between neigboring states*

GLH:13

Gon:4

G23:3

*"x-positions of minima of free energy diagrams*

x1:9

x2:1.5

x3:0

*"Cross-bridge stiffness*

k:2.8

"End of program

end

--------------------------------------------------------------------------------------

| **Parameter** | **30^o^C** | **15^o^C** | **Comments** |
| --- | --- | --- | --- |
| $\Delta$G_AM*DL-_  _AM´DH_ ≡ **$\Delta$G_LH_**  (AM*D_L_- AM*D_H_) | 13 k_B_T | 8 k_B_T | Approximated based on changes in average cross-bridge strain during isometric contraction (Linari et al., 2007) |
| k_+3_ + k_-3_  (Recovery stroke+hydrolysis) | 220 s^-1^ | 40 s^-1^ | Based on data from (Sleep et al., 2005; Offer and Ranatunga, 2015) and references therein |
| K_3_ | 10 | 6 | Based on data from (Sleep et al., 2005; Offer and Ranatunga, 2015) and references therein |
| k_on_´ | 490 s^-1^ | 187 s^-1^ | Based on data from (Sleep et al., 2005; Offer and Ranatunga, 2015) and references therein |
| k_2_ | 2000 s^-1^ | 776s^-1^ | (Nyitrai et al., 2006) |
| k_P+_´ | 10000 s^-1^ | 2240 s^-1^ | As in (Månsson, 2021) |

**Table S3**. Changes in parameter values^a^ for simulation of contractile properties of fast mammalian muscle at 15 ^o^C using starting parameter values for 30 ^o^C as in main Fig. 6F.

^a^Starting parameter values at 30 ^o^C as in Tables S1-S2 except for changes as described in the main Fig. 6F. Parameter values not given here are assumed identical to those in Tables S1-S2 or in Fig. 6F. See (Rahman et al., 2018) for approach to adapt parameter values to lower temperature.

**Supplementary Results**

**Fig. S1. Simulated data for isometric force (*F*) and ATPase compared to experimental data. A.** Simulated force (filled red circles) and ATPase data (open red circles) for 30 ^o^C conditions (parameter values in Table S1, S2 and main Fig. 6F) compared to force and ATPase data from skinned rabbit psoas myofibrils (dashed line (Tesi et al., 2002) and muscle fibers (purple triangles (Potma et al., 1995) or purple circles (Caremani et al., 2008)) at 12-15 ^o^C. **B.** Same experimental data as in A but compared to simulation results for force and ATPase set to mimic conditions at 15 ^o^C (see Table S3) and main Fig. 6F. Note appreciable variability in effects of varied [Pi] on force between different experiments. Also note, maintained difference in effects of varied [Pi] on *F* and *ATPase* in simulations when parameter values are modified from the 30 ^o^C condition (A) to values mimicking 15 ^o^C conditions (B). **C.** Simulated and experimental force data from A replotted without ATPase data for clarity. Same color and symbol coding as in A. **D.** Simulated and experimental force data from B replotted without ATPase data for clarity. Same color and symbol coding as in B. **E.** Simulated and experimental ATPase data from A replotted without force data for clarity. Same color and symbol coding as in A. **F.** Simulated and experimental ATPase data from B replotted without force data for clarity. Same color and symbol coding as in B.

**Supplementary References**

Caremani, M., Dantzig, J., Goldman, Y.E., Lombardi, V., and Linari, M. (2008). Effect of inorganic phosphate on the force and number of myosin cross-bridges during the isometric contraction of permeabilized muscle fibers from rabbit psoas. *Biophys J* 95(12)**,** 5798-5808. doi: S0006-3495(08)81996-0 [pii] 10.1529/biophysj.108.130435.

Elmqvist, H. (1975). *Simnon: An Interactive Simulation Program for Nonlinear Systems : User's Manual.* Lund, Sweden: Department of Automatic Control, Lund Institute of Technology, Institutionen för Reglerteknik.

Linari, M., Caremani, M., Piperio, C., Brandt, P., and Lombardi, V. (2007). Stiffness and fraction of Myosin motors responsible for active force in permeabilized muscle fibers from rabbit psoas. *Biophys J* 92(7)**,** 2476-2490. doi: S0006-3495(07)71052-4 [pii] 10.1529/biophysj.106.099549.

Moretto, L., Usaj, M., Matusovsky, O., Rassier, D.E., Friedman, R., and Månsson, A. (2022). Multistep orthophosphate release tunes actomyosin energy transduction. *Nat Commun* 13(1)**,** 4575. doi: 10.1038/s41467-022-32110-9.

Månsson, A. (2016). Actomyosin based contraction: one mechanokinetic model from single molecules to muscle? *J Muscle Res Cell Motil* 37(6)**,** 181-194. doi: 10.1007/s10974-016-9458-0.

Månsson, A. (2020). Hypothesis: Single Actomyosin Properties Account for Ensemble Behavior in Active Muscle Shortening and Isometric Contraction. *Int J Mol Sci* 21(21). doi: 10.3390/ijms21218399.

Månsson, A. (2021). The effects of inorganic phosphate on muscle force development and energetics: challenges in modelling related to experimental uncertainties. *J Muscle Res Cell Motil* 44**,** 33-46. doi: 10.1007/s10974-019-09558-2.

Månsson, A. (2025a). Theoretical treatment of tension transients in muscle following sudden changes in orthophosphate concentration: implications for energy transduction. *Journal of Muscle Research and Cell Motility* In press. doi: 10.1007/s10974-025-09698-8.

Månsson, A. (2025b). Theoretical treatment of tension transients in muscle following sudden changes in orthophosphate concentration: implications for energy transduction. *J Muscle Res Cell Motil*. doi: 10.1007/s10974-025-09698-8.

Månsson, A., and Rassier, D.E. (2022). Insights into Muscle Contraction Derived from the Effects of Small-Molecular Actomyosin-Modulating Compounds. *Int J Mol Sci* 23(20). doi: 10.3390/ijms232012084.

Nyitrai, M., Rossi, R., Adamek, N., Pellegrino, M.A., Bottinelli, R., and Geeves, M.A. (2006). What limits the velocity of fast-skeletal muscle contraction in mammals? *Journal of Molecular Biology* 355(3)**,** 432-442.

Offer, G., and Ranatunga, K.W. (2015). The endothermic ATP hydrolysis and crossbridge attachment steps drive the increase of force with temperature in isometric and shortening muscle. *J Physiol* 593(8)**,** 1997-2016. doi: 10.1113/jphysiol.2014.284992.

Potma, E.J., van Graas, I.A., and Stienen, G.J. (1995). Influence of inorganic phosphate and pH on ATP utilization in fast and slow skeletal muscle fibers. *Biophys J* 69(6)**,** 2580-2589.

Rahman, M.A., Usaj, M., Rassier, D.E., and Månsson, A. (2018). Blebbistatin Effects Expose Hidden Secrets in the Force-Generating Cycle of Actin and Myosin. *Biophys J* 115(2)**,** 386-397. doi: 10.1016/j.bpj.2018.05.037.

Sleep, J., Irving, M., and Burton, K. (2005). The ATP hydrolysis and phosphate release steps control the time course of force development in rabbit skeletal muscle. *J Physiol* 563(Pt 3)**,** 671-687. doi: 10.1113/jphysiol.2004.078873.

Tesi, C., Colomo, F., Piroddi, N., and Poggesi, C. (2002). Characterization of the cross-bridge force-generating step using inorganic phosphate and BDM in myofibrils from rabbit skeletal muscles. *J Physiol* 541(Pt 1)**,** 187-199.
